# Supplementary material for: Evaluation of the operation status of the residential land market in Beijing-Tianjin-Hebei region of China and its spatiotemporal pattern characteristics
Source: PLoS One. 2023 Sep 21;18(9):e0291648. doi: 10.1371/journal.pone.0291648 (PMC10513223; doi:10.1371/journal.pone.0291648)
Supplement: Attachment — Submitted filename: Response to Reviewers.docx [file pone.0291648.s001.docx]

*We've checked your submission and before we can proceed, we need you to address the following issues:*

*1. We are reaching back out regarding our copyright query. We note that you have not provided a response to our previous attempts to contact you regarding our ongoing concern. Please be aware that if this cannot be resolved we cannot proceed to publication for your manuscript.*

Reply: Thank you very much for your outstanding and valuable suggestion. According to your request, we have deleted controversial images and made all the remaining images by ourselves. The updated images are named fig1a. tif. fig1b.tif. fig1c.tif. fig1d.tif. fig2.tif. fig3a.tif. fig3b.tif. fig3c.tif. fig3d.tif. fig3e.tif. fig3f.tif. fig3g.tif. fig3h.tif. fig4a.tif. fig4b.tif. And corresponding modifications have been made in the manuscript. If you believe that the requirements are not met, we will delete all the images and present the calculation results in a table format.
